# ddPCR: a more accurate tool for SARS-CoV-2 detection in low viral load specimens

**DOI:** 10.1080/22221751.2020.1772678

**Published:** 2020-06-07

**Authors:** Tao Suo, Xinjin Liu, Jiangpeng Feng, Ming Guo, Wenjia Hu, Dong Guo, Hafiz Ullah, Yang Yang, Qiuhan Zhang, Xin Wang, Muhanmmad Sajid, Zhixiang Huang, Liping Deng, Tielong Chen, Fang Liu, Ke Xu, Yuan Liu, Qi Zhang, Yingle Liu, Yong Xiong, Guozhong Chen, Ke Lan, Yu Chen

**Affiliations:** a State Key Laboratory of Virology, Renmin Hospital, Wuhan University, Wuhan, People’s Republic of China; b State Key Laboratory of Virology, Modern Virology Research Center, College of Life Sciences, Wuhan University, Wuhan, People’s Republic of China; c Department of Infectious Disease, Zhongnan Hospital, Wuhan University, Wuhan, People’s Republic of China; d Frontier Science Center for Immunology and Metabolism, Wuhan University, Wuhan, People’s Republic of China

**Keywords:** SARS-CoV-2, droplet digital PCR, RT-PCR, clinical detection, false negative

## Abstract

Quantitative real time PCR (RT-PCR) is widely used as the gold standard for clinical detection of SARS-CoV-2. However, due to the low viral load specimens and the limitations of RT-PCR, significant numbers of false negative reports are inevitable, which results in failure to timely diagnose, cut off transmission, and assess discharge criteria. To improve this situation, an optimized droplet digital PCR (ddPCR) was used for detection of SARS-CoV-2, which showed that the limit of detection of ddPCR is significantly lower than that of RT-PCR. We further explored the feasibility of ddPCR to detect SARS-CoV-2 RNA from 77 patients, and compared with RT-PCR in terms of the diagnostic accuracy based on the results of follow-up survey. 26 patients of COVID-19 with negative RT-PCR reports were reported as positive by ddPCR. The sensitivity, specificity, PPV, NPV, negative likelihood ratio (NLR) and accuracy were improved from 40% (95% CI: 27–55%), 100% (95% CI: 54–100%), 100%, 16% (95% CI: 13–19%), 0.6 (95% CI: 0.48–0.75) and 47% (95% CI: 33–60%) for RT-PCR to 94% (95% CI: 83–99%), 100% (95% CI: 48–100%), 100%, 63% (95% CI: 36–83%), 0.06 (95% CI: 0.02–0.18), and 95% (95% CI: 84–99%) for ddPCR, respectively. Moreover, 6/14 (42.9%) convalescents were detected as positive by ddPCR at 5–12 days post discharge. Overall, ddPCR shows superiority for clinical diagnosis of SARS-CoV-2 to reduce the false negative reports, which could be a powerful complement to the RT-PCR.

## Introduction

The pandemic of coronavirus disease 2019 (COVID-19) caused by the infection of severe acute respiratory syndrome coronavirus 2 (SARS-CoV-2, also refers as HCOV-19) poses a great threat to public health worldwide [1,2]. It presents a huge challenge for the diagnosis of this pathogen. According to World Health Organization (WHO) and Chinese Center for Disease Control and Prevention (CDC), the current gold standard for the diagnosis of SARS-CoV-2 infection is based on the real-time fluorescent quantitative PCR (RT-PCR), which can detect the nucleic acid of SARS-CoV-2 from patient’s specimen [3,4]. The advantages of RT-PCR method are high throughput and relatively sensitive. However, it has been found in clinical practice that some patients had fever and showed symptoms of suspected viral pneumonia such as lower lobe lesions of the lungs by chest computed tomography (CT), but the nucleic acid test of throat swab using RT-PCR did not show positive results until 5–6 days after the onset of viral pneumonia. Remarkably, it was reported that around 60% of SARS-CoV-2 infections are asymptomatic [5]. Moreover, it was estimated that only 30%−60% positive results can be obtained among COVID-19 patients that further confirmed by chest CT and other diagnostic aid [6]. This might be explained by the relatively low viral load in the throat swabs of patients and the limitations of RT-PCR technology, which is easily affected by sample inhibitors, poor amplification efficiency, less precision in low-concentration samples, the subjective cut-off values and the quantification depending on a calibration curve [7–9]. Therefore, RT-PCR inevitably produced false negatives during the clinical diagnosis, leading to a potential risk of viral transmission. Moreover, supposed convalescents, who are about to discharge, especially need viral nucleic acid test with true negative results for confirmation and the out of quarantine to avoid virus transmission and recurrence. Therefore, it is an urgent need for a more sensitive and accurate detection method for the pathogenic detection complementary to current ones.

Digital PCR is based on the principles of limited dilution, end-point PCR, and Poisson statistics, with absolute quantification as its heart [10]. The sample is randomly distributed into discrete partitions (thousands of droplets), some of which contain no template and others contain one or more templates. The partitions are amplified to end point and then counted by a droplet reader to determine the number of positive partitions, from which the concentration is estimated by modelling as a Poisson distribution. Therefore, quantification is less affected by poor amplification efficiency and inhibitors of amplification that may present in samples. The process of sample partitioning also effectively concentrates template molecules within the micro reactions, improving analytical sensitivity for rare species by reducing competition between different targets for amplification reagents in the reaction mixture [11–13]. In 2011, Hindson developed the droplet digital PCR (ddPCR) technology based on traditional digital PCR [14]. The reaction mixture can be divided into tens of thousands of nanodroplets during the process. These vast and highly consistent oil droplets substantially improve the detection dynamic range and accuracy of digital PCR in a low-cost and practical format [15]. In recent years, this technology has been widely used, such as analysis of absolute viral load from clinical samples, analysis of gene copy number variation, rare allele detection, gene expression, microRNA analysis and genome edit detection [13–17].

To improve the diagnostic accuracy of nucleic acid detection of SARS-Cov-2 in low viral load samples using droplet digital PCR, we compared the dynamic range and the limit of detection (LoD) with a 95% repeatable probability between ddPCR and RT-PCR in laboratory, and tested the clinical samples from 77 patients by both ddPCR and RT-PCR for head to head comparison.

## Materials and methods

### Ethics statement

The institutional review board of Renmin Hospital of Wuhan University approved this study (WDRY2020-K089). Written informed consents were obtained.

### Specimen collection

Data collection were planned before the index test and reference standard were performed (prospective study). To perform the tests of clinical samples, throat swab samples of 63 suspected outpatients and 14 supposed convalescents were collected by the medical staffs from COVID-19 designated Renmin and Zhongnan Hospital of Wuhan University. Throat swab samples of each patient were firstly collected for official approved RT-PCR diagnosis in hospitals and blinding laboratory RT-PCR and ddPCR tests simultaneously with the same primers/probe sets approved by Chinese CDC. The patients were conducted by hospitals independently. All the events happened in hospitals and laboratories are blinded to each other during the tests. The follow-up survey and clinical information of enrolled cohort were collected after the laboratory tests by the medical staffs.

### RNA extraction

Throat swab samples were collected via mouth according to the interim guidance of WHO, and soaked in 500 μl PBS and vortexed with diameter of 3 mm beads (Novastar, China) for 15 s immediately. Total RNA was extracted from the supernatant using QIAamp viral RNA mini kit (Qiagen) following manufacturer’s instruction. First strand cDNA was synthesized using PrimeScript RT Master Mix (TakaRa) with random primer and oligo dT primer for subsequent tests of both RT-PCR and ddPCR in laboratory simultaneously.

### Primers and probes

The primers and probes (RainSure Scientific) target the ORF1ab and N of SARS-CoV-2 according to Chinese CDC.

Target 1 (ORF1ab), forward: 5′-CCCTGTGGGTTTTACACTTAA-3′,

reverse: 5′-ACGATTGTGCATCAGCTGA-3′,

probe: 5′-FAM-CCGTCTGCGGTATGTGGAAAGGTTATGG-BHQ1–3′;

Target 2 (N), forward: 5′-GGGGAACTTCTCCTGCTAGAAT-3′,

reverse: 5′-CAGACATTTTGCTCTCAAGCTG-3′,

probe: 5′-HEX-TTGCTGCTGCTTGACAGATT-TAMRA-3′ [18].

### Droplet digital PCR workflow

All the procedures follow the manufacture instructions of the QX200 Droplet Digital PCR System using supermix for probe (no dUTP) (Bio-Rad). Briefly, the TaqMan PCR reaction mixture was assembled from 2× supermix for probe (no dUTP) (Bio-Rad), 20× primers and probe mix (final concentrations of 900 and 250 nM, respectively) and template (variable volume, cDNA of clinic sample or linear DNA standard) in a final volume of 20 μl. Twenty microliters of each reaction mix was converted to droplets with the QX200 droplet generator (Bio-Rad). Droplet-partitioned samples were then transferred to a 96-well plate, sealed and cycled in a T100 Thermal Cycler (Bio-Rad) under the following cycling protocol: 95°C for 10 min (DNA polymerase activation), followed by 40 cycles of 94°C for 30 s (denaturation) and 60°C for 1 min (annealing) followed by an infinite 4-degree hold. The cycled plate was then transferred and read in the FAM and HEX channels using the QX200 reader (Bio-Rad). To avoid the risk of viral infection and false positive results potentially due to the laboratory contamination, all the experiments were done inside the biosafety cabinet in negative pressure biosafety laboratory using filter tips.

### RT-PCR

The primers and probes used in ddPCR are also used in the RT-PCR system established in laboratory. A 20 μl reaction mix was set up containing 8 μl of template (variable volume, cDNA of clinic sample or linear DNA standard), 10 μl of reaction buffer, 1 μl 20× primers and probe mix and 1 µl Platinum Taq DNA Polymerase mix (Thermo fisher). Thermal cycling was performed at 95°C for 5 min and then 40 cycles of 95°C for 10 s, 55°C for 40 s in BIO-RAD CFX96 Touch Real-Time PCR Detection system (Bio-Rad). To avoid the risk of viral infection and laboratory contamination, the same biosafety measures were taken as that for ddPCR.

### Dynamic range and LoD of RT-PCR and ddPCR

The linear dynamic range of the RT-PCR and ddPCR assay was assessed using the serial dilutions of the linear DNA standard containing the target region. To determine the LoD of both RT-PCR and ddPCR, cDNA of throat swab samples of healthy people was spiked with the linear DNA standard in serial concentrations close to the detection limits. The LoD was calculated by Probit regression analysis with a 95% repeatable probability, which is a commonly used type of regression analysis when empirically determining the limit of analyte that can be reliably detected by molecular assays [19].

### Data statistical analysis

Analysis of the ddPCR data were performed with Quanta Soft analysis software v.1.7.4.0917 (Bio-Rad) to calculate the concentration of the target. The positive populations for each primer/probe are identified using positive and negative controls with single (i.e. not multiplexed) primer–probe sets. In addition, plots of linear regression were conducted with GraphPad Prism 7.00, and probit analysis for LoD was conducted with MedCalc software v19.2.1.

The cases of lost contact were not included in our analysis study due to the unclear conditions. The suspected reports of ddPCR (need further detection) were not included in our analysis study according to the ethics statement as no more sampling for laboratory tests. The detection results were compared to the follow-up survey including clinical records of chest computed tomography, IgM/IgG, other aetiological detection, and further official approved RT-PCR confirmation 2–12 days later. The diagnostic performance of RT–PCR and ddPCR were calculated by MEDCALC (https://www.medcalc.org/calc/diagnostic_test.php).

## Results

### Comparison of the dynamic range of ddPCR and RT-PCR

**Figure 1.**
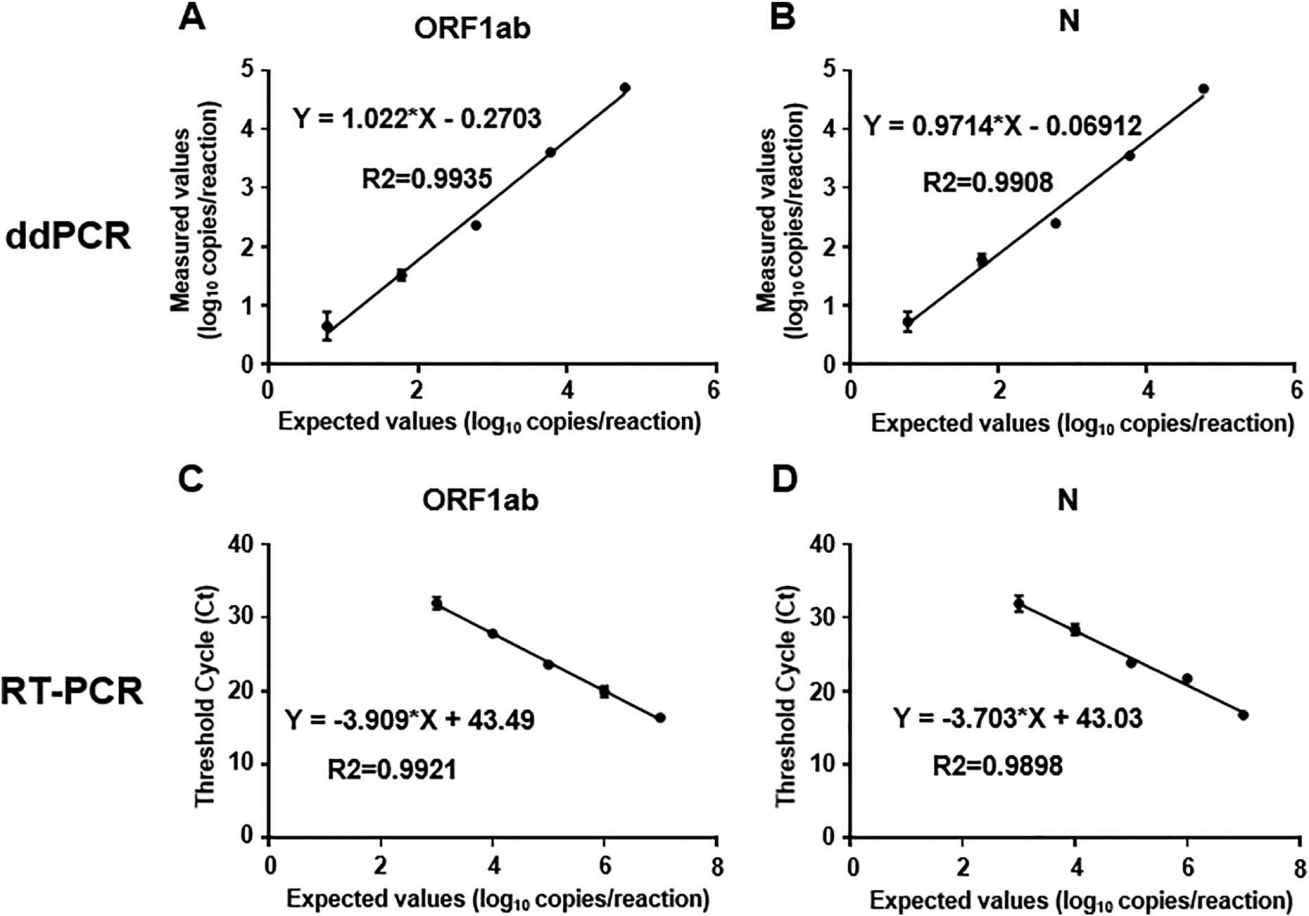
Plot of results from a linearity experiment to determine the reportable range of ddPCR and RT-PCR targeting ORF1ab and N of SARS-CoV-2. (A and B) Expected values (converted to log_10_) were plotted on the *X*-axis versus measured values of ddPCR (converted to log_10_) on the *Y*-axis using Graph Pad Prism targeting (A) ORF1ab and (B) N. (C and D) Expected values (converted to log_10_) were plotted on the *X*-axis versus measured Ct values of RT-PCR on the *Y*-axis using Graph Pad Prism targeting (C) ORF1ab and (D) N. Data are representative of three independent experiments with 3 replicates for each concentration (means ± SD).

To compare the dynamic range of ddPCR and RT-PCR, serial dilutions of a positive control linear DNA standard of SARS-CoV-2 were tested using primers/probe sets targeting ORF1ab and N of SARS-CoV-2 for both ddPCR and RT–PCR. As shown in Figure 1, the reportable range of ddPCR is 10–5 × 10^4^ copies/reaction for both ORF1ab and N primes/probe sets with R2 = 0.9935 and 0.9908, respectively (Figure 1(A,B)). Meanwhile, those of RT-PCR is 1000–10^7^ copies/reaction for both ORF1ab and N primes/probe sets with R2 = 0.9921 and 0.9898, respectively (Figure 1(C,D)). The results showed that the minimum detection range of ddPCR is significantly lower than that of RT-PCR.

### Comparison of the LoD between ddPCR and RT-PCR

**Figure 2.**
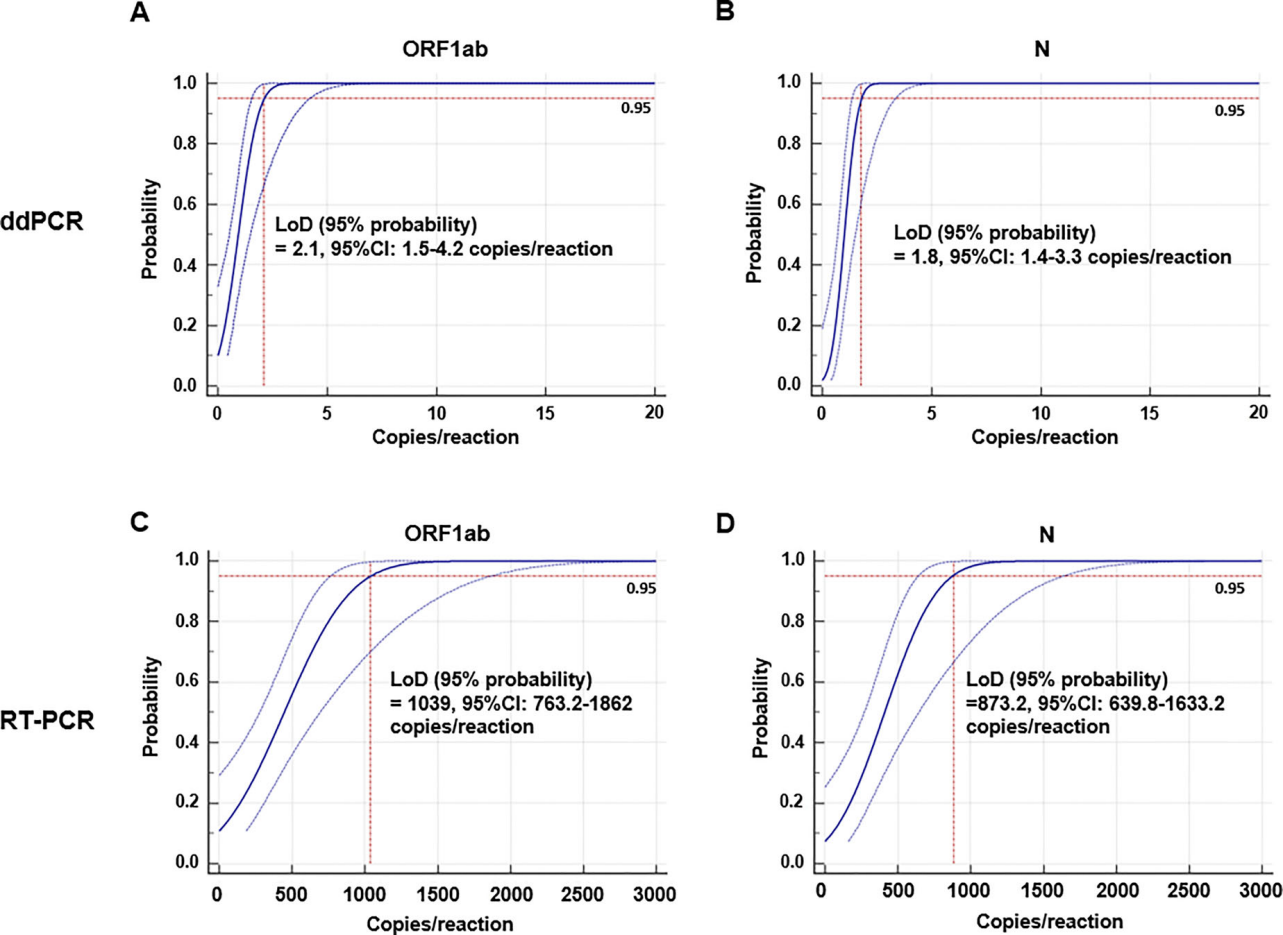
Probit analysis sigmoid curve reporting the LoD of ddPCR and RT-PCR. Replicate reactions of (A) ORF1ab and (B) N of ddPCR or (C) ORF1ab and (D) N of RT-PCR were done at concentrations around the detection end point determined in preliminary dilution experiments. The *X*-axis shows expected concentration (copies/reaction). The *Y*-axis shows fraction of positive results in all parallel reactions performed. The inner line is a probit curve (dose-response rule). The outer lines are 95% confidence interval (95% CI). Data are representative of three independent experiments with 8 replicates for each concentration.

To further determine the accurate LoD of ddPCR and RT-PCR, a series linear DNA standard were diluted to the concentrations below the minimum detection range of ddPCR or RT-PCR by the cDNA of throat swab samples from healthy people (with negative serum SARS-CoV-2 IgM/IgG), which could benefit to reduce the false positive result (FPR). Each concentration was analysed with eight replicates. The LoD was calculated by probit regression with a 95% repeatable probability. As shown in Figure 2, the LoD (95% probability) of ddPCR are 2.1 (95% CI: 1.5–4.2) copies/reaction and 1.8 (95% CI: 1.4–3.3) copies/reaction for ORF1ab (Figure 2(A)) and N (Figure 2(B)) primers/probe sets, respectively. In contrast, the LoD (95% probability) of RT-PCR are 1039 (95% CI: 763.2–1862) copies/reaction and 873.2 (95% CI: 639.8–1633.2) copies/reaction for ORF1ab (Figure 2(C)) and N (Figure 2(D)) primers/probe sets, respectively. Taken together, with the same ORF1ab and N primes/probe sets and template, ddPCR for SARS-CoV-2 detection with a 95% probability, is around 500 times (maximum) more sensitive than RT-PCR in low level analyte.

### Detection of SARS-CoV-2 nucleic acid from patient throat swab samples with ddPCR and RT-PCR

To avoid the FPR potentially due to the laboratory contamination, all the experiments were performed inside the biosafety cabinet in negative pressure biosafety laboratory using filter tips. To further assess the systematic FPR, the cDNA of throat swab samples from healthy people were tested with ddPCR for 32 repeats (16 repeats for each primers/probe set, data not shown). In total, 29 out of 32 results (91%) showed negative read out (0 copy/reaction). Three out of 32 results (9%) showed one single positive droplet (around 1 copy/reaction), which is less than the LoD of ddPCR for ORF1ab (2.1 copies/reaction) and N (1.8 copies/reaction) primers/probe sets. Therefore, the positive threshold of ddPCR for SARS-CoV-2 detection is defined as equal as or greater than the LoD of ddPCR for ORF1ab and N primers/probe sets, respectively. The result between 0 copy/reaction and the LoD of ddPCR for each primers/probe sets are defined as suspected SARS-CoV-2 infection, which needs further detection. The outcome 0 copy/reaction for both ORF1ab and N primers/probe sets is judged as negative.

**Figure 3.**
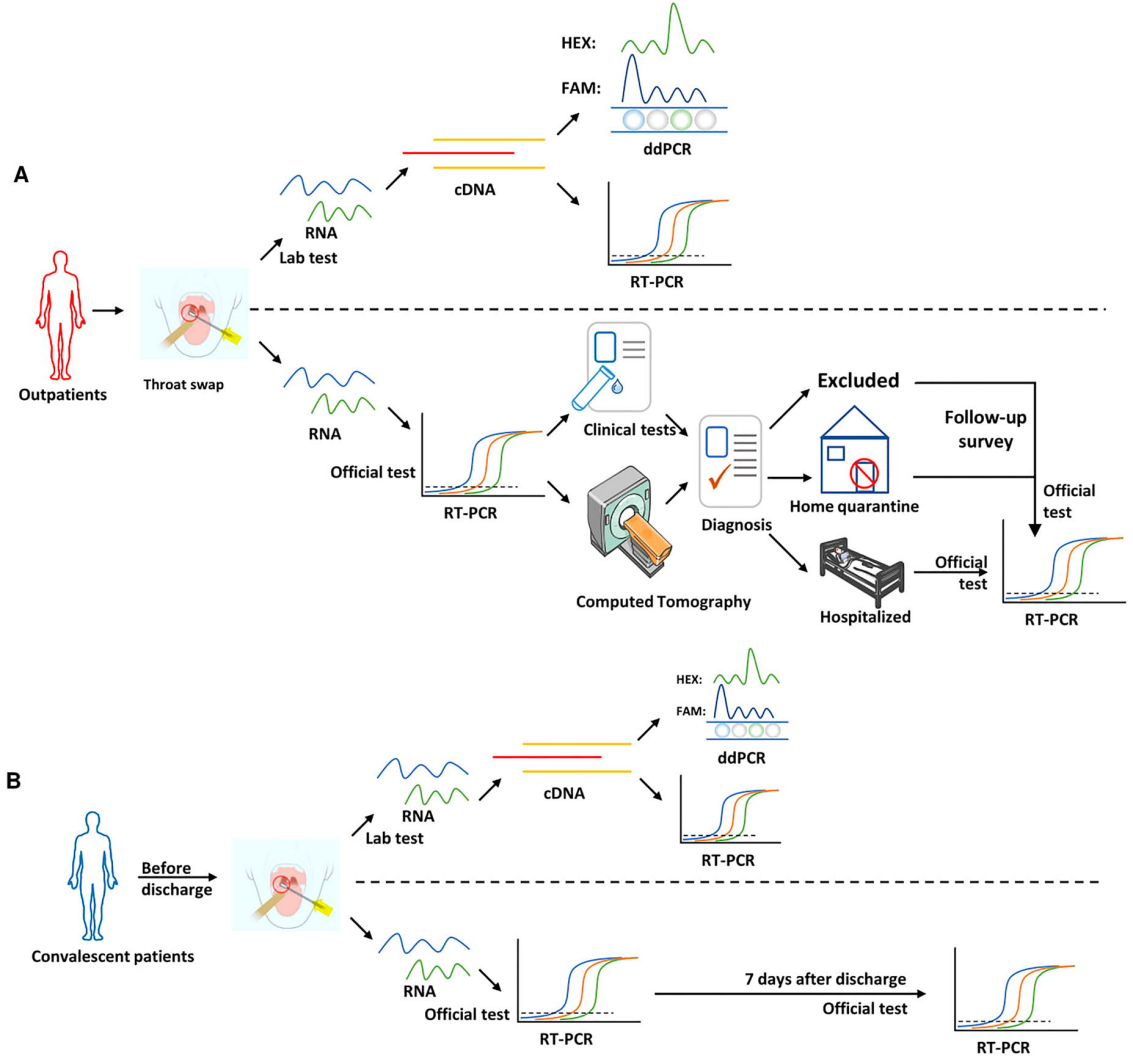
Flowchart of this research design. (A) Research design for suspected outpatients and (B) supposed convalescents. These results were acquired in blind from hospitals and laboratory independently. The official approved RT-PCR were conducted by hospitals. The follow-up survey and clinical information of enrolled patients were used to evaluate the performance of ddPCR and RT-PCR.

As shown in Figure 3, throat swab samples of each suspected outpatient were firstly collected for laboratory RT-PCR, ddPCR tests and official approved RT-PCR diagnosis in hospitals simultaneously with the same primers/probe sets approved by Chinese CDC (Table 1). Then the suspected outpatients were diagnosed with chest CT. Based on the official medical programme of China, outpatients with either official approved RT-PCR (positive) or chest CT (ground glass opacities image, GGO) should be hospitalized. Subsequently, the throat swab samples of all hospitalized patients were collected again and subjected to an official approved RT-PCR test at indicated days post hospitalized (Table 1) to monitor the viral load continuously based on the official medical programme.

**Table 1.** RT-PCR and ddPCR Results of suspected outpatients of COVID-19 and their further clinical information.

Series No.	Results of RT-PCR in lab (Ct value)^a,b^		Result of ddPCR (copies/reaction)^a,b^		Judgment by ddPCR	Initial official reports by RT-PCR^a^	Further diagnosis by chest CT^c^	Patient condition^d^	Official reports by RT-PCR again after (days)^e^
	ORF1ab	N	ORF1ab	N					
P1	ND	ND	0	2.0	P	N	GGO^c^	Hospitalized	P (4d)
P2	39.46	38.90	0	2.0	P	N	GGO	Hospitalized	P (4d)
P3	ND	ND	0	2.0	P	N	GGO	Hospitalized	P (4d)
P4	35.20	35.98	4.8	20.8	P	P	GGO	Hospitalized	P (6d)
P5	ND	39.59	0	3.6	P	N	GGO	Hospitalized	P (4d)
P6	36.24	35.45	32.4	104	P	P	GGO	Hospitalized	P (6d)
P7	ND	ND	3.4	13.6	P	N	GGO	Hospitalized	P (2d)
P8	36.87	36.10	18.6	36.4	P	P	GGO	Hospitalized	P (6d)
P9	39.19	38.87	1.6	13.2	P	N	GGO	Hospitalized	P (3d)
P10	ND	ND	0	1.6	S	N	GGO	Hospitalized	P (4d)
P11	35.08	33.52	4.8	16.4	P	P	GGO	Hospitalized	P (6d)
P12	ND	ND	0	3.6	P	N	GGO	Hospitalized	P (4d)
P13	ND	ND	0	4.6	P	N	GGO	Hospitalized	P (4d)
P14	38.09	37.63	2.6	3.8	P	N	GGO	Hospitalized	P (2d)
P15	ND	ND	0	3.6	P	N	GGO	Hospitalized	P (2d)
P16	ND	ND	0	1.8	P	N	GGO	Hospitalized	P (4d)
P17	33.97	32.76	24.4	60.8	P	P	GGO	Hospitalized	P (6d)
P18	ND	ND	0	7.4	P	N	GGO	Hospitalized	P (2d)
P19	ND	ND	0	1.8	P	N	GGO	Hospitalized	P (4d)
P20	37.77	36.63	16.2	36.2	P	P	GGO	Hospitalized	P (6d)
P21	ND	ND	3.8	1.8	P	N	GGO	Hospitalized	P (3d)
P22	ND	ND	2.4	0	P	N	GGO	Hospitalized	P (4d)
P23	32.19	31.81	108	132	P	P	GGO	Hospitalized	P (6d)
P24	28.02	27.49	1130	4440	P	P	GGO	Hospitalized	P (6d)
P25	ND	ND	0	1.8	P	N	GGO	Hospitalized	P (4d)
P26	ND	ND	0	1.4	S	N	GGO	Hospitalized	P (5d)
P27	25.33	24.25	544	1384	P	P	GGO	Hospitalized	P (6d)
P28	27.02	26.30	22.8	98.2	P	P	GGO	Hospitalized	P (6d)
P29	ND	ND	0	2.2	P	N	GGO	Hospitalized	P (10d)
P30	36.15	36.02	26.2	68.4	P	P	GGO	Hospitalized	P (6d)
P31	ND	ND	0	6.6	P	N	GGO	Hospitalized	P (8d)
P32	34.93	33.52	32.6	64.4	P	P	GGO	Hospitalized	P (6d)
P33	ND	ND	4.4	14.2	P	N	GGO	Hospitalized	P (7d)
P34	28.98	28.69	746	1954	P	P	GGO	Hospitalized	P (6d)
P35	ND	39.46	0	3.2	P	N	GGO	Hospitalized	P (4d)
P36	31.49	30.63	36.2	140	P	P	GGO	Hospitalized	P (6d)
P37	28.22	27.26	20.8	40.4	P	P	GGO	Hospitalized	P (6d)
P38	27.09	26.48	8.6	36.2	P	P	GGO	Hospitalized	P (6d)
P39	ND	ND	0	1.8	P	N	GGO	Hospitalized	P (8d)
P40	ND	ND	0	3.2	P	N	GGO	Hospitalized	P (8d)
P41	32.73	33.18	16.2	11.4	P	P	GGO	Hospitalized	P (6d)
P42	34.16	33.09	22.4	32.8	P	P	GGO	Hospitalized	P (6d)
P43	25.02	27.30	1.8	2.4	P	P	GGO	Hospitalized	P (6d)
P44	37.01	36.57	16.6	28.4	P	P	GGO	Hospitalized	P (6d)
P45	35.85	35.26	10.2	32.2	P	P	GGO	Hospitalized	P (6d)
P46	ND	ND	0	0	N	N	GGO	Hospitalized	P (6d)
P47	ND	ND	0	0	N	N	GGO	Hospitalized	P (8d)
P48	ND	39.27	0	3.4	P	N	Pleural bleb	Home Quarantine	NA, lose contact
P49	ND	ND	0	1.2	S	N	Lower-lobe pneumonia	Home Quarantine	NA, lose contact
P50	ND	ND	1.6	4.0	P	N	Pneumonia	Home Quarantine	P (10d)
P51	ND	38.83	0	3.8	P	N	Secondary pulmonary tuberculosis	Home Quarantine	P (9d)
P52	39.83	38.51	0	5.6	P	N	Normal	Home Quarantine	NA, lose contact
P53	39.60	38.16	3.0	16.2	P	N	Fibrous stripes	Home Quarantine	P (7d)
P54	ND	ND	0	2.0	P	N	Subpleural nodules	Home Quarantine	P (11d)
P55	ND	ND	0	0	N	N	Normal	Home Quarantine	Negative (4, 6 and 8d), asymptomatic
P56	ND	ND	0	1.2	S	N	Normal	Excluded	N (6d)
P57	ND	ND	0	0	N	N	Emphysema	Excluded	NA, lose contact
P58	ND	ND	0	0	N	N	Fibrous stripes	Excluded	N (6d)
P59	ND	ND	0	0	N	N	Normal	Excluded	N (5d)
P60	ND	ND	0	0	N	N	Normal	Excluded	NA, lose contact
P61	ND	ND	0	0	N	N	Pneumonia	Excluded	N (6d)
P62	ND	ND	0	0	N	N	Fibrous stripes	Excluded	N (6d)
P63	ND	ND	0	0	N	N	Nodules	Excluded	N (8d)

Note: P, positive; N, Negative; S, suspect; ND, not detected; NA, not applicable.

^a^Throat swab samples were firstly collected for laboratory RT-PCR, ddPCR tests and official approved RT-PCR diagnosis in hospitals simultaneously using the same primers/probe sets approved by Chinese CDC. In laboratory tests, the RNA of throat swab samples were extracted and reverse transcribed to cDNA that is subjected to both RT-PCR and ddPCR tests subsequently.

^b^The reaction systems of laboratory RT-PCR and ddPCR are 20 μl/reaction.

^c^The subsequent results of chest CT were used to diagnose the COVID-19 in hospitals based on the official medical programme of China. GGO, ground glass opacities image.

^d^Outpatients with chest CT (GGO) or official approved RT-PCR (positive) were hospitalized. Outpatients with chest CT (no GGO), official approved RT-PCR (negative) but ddPCR (positive) were suggested to be quarantined at home. Outpatients with CT (normal), official approved RT-PCR (negative), ddPCR (negative) and other supportive clinical tests (supplementary Table S1) were excluded for COVID-19.

^e^The infection of SARS-CoV-2 was confirmed by official approved RT-PCR test after the indicated days.

The supposed convalescents should be that: (1) temperature returned to normal for more than 3 days, and respiratory symptoms significantly improved; (2) chest CT imaging showed significant absorption of inflammation; (3) the nucleic acid test of respiratory pathogen was negative for two consecutive times, and the sampling interval should be at least one day, based on the official medical programme. In this study, two throat swab samples of each supposed convalescent were collected for laboratory RT-PCR, ddPCR and official approved RT-PCR tests simultaneously with the same primers/probe sets (Table 2). Subsequently, the throat swab samples of discharged convalescents were collected again and subjected to official approved RT-PCR test at indicated days post discharge to assess the viral load after discharge (Table 2).

**Table 2.** Results of RT-PCR and ddPCR for supposed convalescents who are about to be discharged after treatments.

Patient Number	Patient status	Official reports by RT-PCR^a^	Results of RT-PCR in lab (Ct Value)^b,c^		Result of ddPCR (copies/reaction)^b,c^		Judgment by ddPCR	Official reports by RT-PCR again after (days)^d^
			ORF1ab	N	ORF1ab	N		
P64	Supposed convalescent	N	ND	ND	0	2.4	P	P (12d)
P65	Supposed convalescent	N	ND	ND	0	2.2	P	P (7d)
P66	Supposed convalescent	N	39.33	39.05	11.4	12.0	P	N (7d)
P67	Supposed convalescent	N	ND	40.07	0	9.0	P	N (6d)
P68	Supposed convalescent	N	40.03	39.82	0	16.0	P	N (7d)
P69	Supposed convalescent	N	ND	ND	1.8	0	S	P (7d)
P70	Supposed convalescent	N	ND	ND	0	2.2	P	P (7d)
P71	Supposed convalescent	N	39.79	38.90	3.8	106	P	P (5d)
P72	Supposed convalescent	N	ND	40.02	1.4	1.4	S	N (7d)
P73	Supposed convalescent	N	ND	ND	0	0	N	N (7d)
P74	Supposed convalescent	N	39.61	ND	0	0	N	N (7d)
P75	Supposed convalescent	N	ND	40.01	0	0	N	N (7d)
P76	Supposed convalescent	N	ND	ND	0	0	N	N (7d)
P77	Supposed convalescent	N	ND	ND	0	0	N	P (7d)

Note: P, positive; N, Negative; S, suspect; ND, not detected.

^a^ Throat swab samples of supposed convalescent were firstly collected for official approved RT-PCR test and laboratory RT-PCR and ddPCR tests.

^b^The collected samples were test for both RT-PCR and ddPCR in laboratory simultaneously using the same reverse transcriptase system and primers/probe sets approved by Chinese CDC.

^c^The reaction systems of laboratory RT-PCR and ddPCR are 20 μl/reaction.

^d^The nucleic acid of SARS-CoV-2 was tested again by official approved RT-PCR after the indicated days to assess the viral load of discharged convalescents.

In the laboratory tests, the RNA of throat swab samples were extracted and reverse transcribed to cDNA that is subjected to both RT-PCR and ddPCR tests. The follow-up survey and clinical information of enrolled cohort were listed in supplementary Table S1 after the laboratory tests.

### Analysis and comparison of the performance of ddPCR and RT-PCR for SARS-CoV-2 diagnosis

Among the 63 suspected outpatients (P1–P63), 21 positive and 42 negative were reported by official approved RT-PCR in two hospitals, which were also double checked by our laboratory RT-PCR (collectively referred to as RT-PCR in performance analysis). In contrast, 49 positive, 10 negative, and 4 suspected SARS-CoV-2 infections were reported by ddPCR according to the above criteria (Table 3). The follow-up survey (Table 1 and supplementary Table S1) revealed that 47 cases (P1–P47) out of 63 were hospitalized subsequently with ground glass opacities images (GGO) of chest CT [20], which were further confirmed as SARS-CoV-2 infection by official approved RT-PCR at 2–10 days post hospitalized. Besides, 7 cases (P48–P54) with ddPCR positive (1 suspected), RT-PCR negative and other images (not GGO) of chest CT were suggested to be quarantined at home considering the positive/suspected reports of ddPCR. The follow-up survey revealed that 4 cases (P50, P51, P53, and P54) out of 7 developed difficulty breathing later, and were confirmed as SARS-CoV-2 infection in other hospital. The rest 3 cases (P48, P49, and P52) out of 7 have lost contact for tracking. Of note, 1 case (P55) with ddPCR negative, RT-PCR negative and normal images of chest CT were SARS-CoV-2 IgM/IgG positive. The further tests by official approved RT-PCR still showed negative reports at 4, 6, and 8 days later, indicating asymptomatic infection of SARS-CoV-2. Meanwhile, 6 out of 8 cases (P56-P63) with ddPCR negative (1 suspected), RT-PCR negative and other images (not GGO) of chest CT were excluded by negative reports of SARS-CoV-2 IgM/IgG (P56, P61, and P62) and official approved RT-PCR tests at 5–8 days later (P56, P58, P59, and P61–P63) in the follow-up survey. Moreover, all of the 6 cases have reported good health. The rest 2 cases (P57 and P60) have lost contact for tracking. We further analysed and compared the performance of RT-PCR and ddPCR for the nucleic acid detection of SARS-CoV-2 according to the follow-up survey and clinical information (Table 4). The 5 cases of lost contact were not included in our analysis study due to the unclear conditions. The sensitivity, specificity, PPV, NPV, NLR, and accuracy were improved from 40% (95% CI: 27–55%), 100% (95% CI: 54–100%), 100%, 16% (95% CI: 13–19%), 0.6 (95% CI: 0.48–0.75), and 47% (95% CI: 33–60%) for RT-PCR to 94% (95% CI: 83–99%), 100% (95% CI: 48–100%), 100%, 63% (95% CI: 36–83%), 0.06 (95% CI: 0.02–0.18), and 95% (95% CI: 84–99%) for ddPCR, respectively.

**Table 3.** Reports summary of RT-PCR and ddPCR for clinical samples compared to the follow-up survey.

	RT-PCR^#^	ddPCR	Follow-up survey	Total of follow-up survey
63 outpatients	21 P	21 P	21 P	52 P 6 N 5 L
	42 N	28 P	26 P	
			2 L	
		10 N	3 P	
			5 N	
			2 L	
		4 S	2 P	
			1 N	
			1 L	
14 supposed convalescents	14 N	7 P	4 P	6 P 8 N
			3 N	
		5 N	1 P	
			4 N	
		2 S	1 P	
			1 N	

Note: N, Negative; P, positive; L, lost contact; S, suspected.

^#^Results of official approved RT-PCR in two hospitals were also checked by laboratory RT-PCR (collectively referred to as RT-PCR).

**Table 4.** Diagnostic performance of ddPCR and RT-PCR for suspect outpatients.

		Clinically positive	Clinically negative	Sensitivity (95% CI)	Specificity (95% CI)	PPV (95% CI)	NPV (95% CI)	Negative likelihood ratio (95% CI)	Accuracy (95% CI)
**RT-PCR**	Test positive	21 ^a^	0^b^	40% (27–55%)	100% (54–100%)	100% (NA)	16% (13–19%)	0.60 (0.48–0.75)	47% (33–60%)
	Test negative	31 ^c^	6^d^						
**ddPCR**	Test positive	47 ^a^	0^b^	94% (83–99%)	100% (48–100%)	100% (NA)	63% (36–83%)	0.06 (0.02–0.18)	95% (84–99%)
	Test negative	3 ^c^	5^d^						
	Suspect	2	1						

Note: Sensitivity = [a/(a + c)] × 100%; Specificity = [d/(b + d)] × 100%; PPV = [a/(a + b)] × 100%, positive predictive value;

NPV = [d / (c + d)] × 100%, negative predictive value; Negative likelihood ratio = (1-Sensitivity)/Specificity;

Accuracy = [(a + d) / (a + b + c + d)] × 100%; NA, not applicable.

Calculated by MEDCALC (https://www.medcalc.org/calc/diagnostic_test.php).

Among the 14 supposed convalescents (P64–P77), whose samples were all reported as negative by official approved RT-PCR, 7 positive, 5 negative, and 2 suspected SARS-CoV-2 infections were reported by ddPCR (Table 3). The follow-up survey (Table 2 and supplementary Table S1) revealed that 5 cases (P64, P65, and P69–P71) out of 9 (P64–P72) with ddPCR positive (2 suspected), RT-PCR negative and lesions absorbed images of chest CT have been diagnosed as SARS-CoV-2 nucleic acid positive again by official approved RT-PCR at 5–12 days post discharge. The rest 4 cases (P66–P68 and P72) out of 9 have remained as nucleic acid negative. Meanwhile, another 4 cases (P73–P76) out of 5 (P73–P77) with ddPCR negative, RT-PCR negative, and lesions absorbed images of chest CT still remained as nucleic acid negative at 7 days post discharge, indicating functional cure. Of note, 1 case (P77) with the same conditions has returned to nucleic acid positive at 7 days post discharge.

## Discussion

More and more nucleic acid detection kits have been developed for SARS-CoV-2 recently based on RT-PCR to meet the requirement of large-scale clinical molecular diagnosis. It has been reported that 6 kinds of RT-PCR detection kits were compared and analysed for their detection performance, which showed that there were differences in the detection ability for weakly positive samples, and the accuracy, sensitivity, and reproducibility of some kits are not ideal [8]. Meanwhile, other methods, such as chest CT and immunological detection of IgM/IgG have been used to help for the diagnosis of COVID-19. However, the direct detection of virus is irreplaceable. Different from RT-PCR that the data are measured from a single amplification curve and a Cq value, which is highly dependent on reaction efficiency, primer dimers, and sample contaminants, ddPCR is measured at reaction end point which virtually eliminates these potential pitfalls.

In this study, we showed that 26 samples from COVID-19 outpatients with RT-PCR negative were detected as positive by ddPCR using the same samples. Accordingly, the NPV of ddPCR (63%, 36–83) is obviously higher than that of RT-PCR (16%, 13–19), which indicates that part of true COVID-19 outpatients (26 positive reports by ddPCR in this study) could not be diagnosed in time by RT–PCR, potentially leading to the higher risk of severe illness and viral spreading (Tables 3 and 4). Remarkably, 4 cases (P50, P51, P53, and P54) with ddPCR positive, RT-PCR negative and other images (not GGO) of chest CT were confirmed as SARS-CoV-2 infection 7–11 days later, indicating that our suggestion of quarantine according to the reports of ddPCR is reasonable. The application of ddPCR for SARS-CoV-2 diagnosis would help to early treatment and control the viral transmission. In conclusion, compared with RT-PCR, ddPCR show superiority for clinical detection of SARS-CoV-2 to reduce the false negatives, which could be a powerful complement to the current standard RT-PCR.

Notably, 6 cases (P64, P65, P69–P71, and P77) out of 14 (42.9%) supposed convalescent patients, who are negative for throat swab nucleic acid tests twice by RT-PCR, are still carrying SARS-CoV-2 according to the follow-up survey. Although the risk of viral transmission is unknown, the virus is replicating, leading to the increase of viral load. Therefore, the current clinical practice that the convalescent continues to be quarantined for at least two weeks is reasonable and necessary. Therefore, we recommend that ddPCR could be a complement to the current standard RT-PCR to re-confirm the convalescent, which would benefit to reduce the risk of the SARS-CoV-2 epidemic and social panic.

However, the specificity and PPV were 100% for both RT-PCR and ddPCR because of no false positives. Partly because the sample size is small, but also the clinical samples we collected were from designated hospitals in Wuhan during the COVID-19 epidemic, which meant the disease prevalence of COVID-19 was higher than common clinical scenarios. Moreover, we used only primers/probes sets from China CDC, which could not represent primers from other official institutes. Further research to compare the efficiency of these different primers needs to be conducted, which helps to improve the diagnostic accuracy of SARS-CoV-2 detection in different countries.

## Supplementary Material

Suppliemtary_Table_S1_EMI_0502.docx
