# Supplementary material for: ddPCR: a more accurate tool for SARS-CoV-2 detection in low viral load specimens
Source: Emerg Microbes Infect. 2020 Jun 7;9(1):1259–68. doi: 10.1080/22221751.2020.1772678 (PMC7448897; doi:10.1080/22221751.2020.1772678)
Supplement: Suppliemtary_Table_S1_EMI_0502.docx [file TEMI_A_1772678_SM9132.docx]

Table S1. Clinical information about all patients.

| Series No. | Further diagnosis by chest CT | Historical epidemiology  of SARS-CoV-2 | Patient condition | White blood cell count, ×10^9^/L  Normal Range  3.5-9.5 | Lymphocyte count, ×10^9^/L  Normal Range  1.1-3.2 | SARS-CoV-2  IgM / IgG  AU/ml  Normal Range  < 10 | Other etiological detection^b^ |
| --- | --- | --- | --- | --- | --- | --- | --- |
| P1 | GGO^a^ | Yes | Hospitalized | 3.47 | 0.93 ↓ | 0.87 / 81.37 ↑ | all negative |
| P2 | GGO | Yes | Hospitalized | 6.63 | 2.33 | NA | all negative |
| P3 | GGO | Yes | Hospitalized | 7.94 | 1.94 | NA | all negative |
| P4 | GGO | Yes | Hospitalized | 5.97 | 2.20 | NA | all negative |
| P5 | GGO | Yes | Hospitalized | 4.91 | 0.68 ↓ | NA | all negative |
| P6 | GGO | Yes | Hospitalized | 6.44 | 2.48 | NA | all negative |
| P7 | GGO | Yes | Hospitalized | 3.40 ↓ | 0.64 ↓ | NA | all negative |
| P8 | GGO | Yes | Hospitalized | 2.70 ↓ | 0.88 ↓ | NA | all negative |
| P9 | GGO | Yes | Hospitalized | 2.72 ↓ | 0.60 ↓ | NA | all negative |
| P10 | GGO | Yes | Hospitalized | 3.31 ↓ | 0.57 ↓ | NA | all negative |
| P11 | GGO | Yes | Hospitalized | 19.24 ↑ | 0.65 ↓ | NA | all negative |
| P12 | GGO | Yes | Hospitalized | 4.69 | 0.89 ↓ | NA | all negative |
| P13 | GGO | Yes | Hospitalized | 4.33 | 1.43 | NA | all negative |
| P14 | GGO | Yes | Hospitalized | 6.56 | 2.22 | NA | all negative |
| P15 | GGO | Yes | Hospitalized | 4.21 | 1.48 | NA | all negative |
| P16 | GGO | Yes | Hospitalized | 4.83 | 1.39 | NA | all negative |
| P17 | GGO | Yes | Hospitalized | 0.90 ↓ | 0.42 ↓ | NA | all negative |
| P18 | GGO | Yes | Hospitalized | 3.11 ↓ | 1.42 | NA | all negative |
| P19 | GGO | Yes | Hospitalized | 3.11 ↓ | 0.76 ↓ | NA | all negative |
| P20 | GGO | Yes | Hospitalized | 9.77 ↑ | 0.40 ↓ | NA | all negative |
| P21 | GGO | Yes | Hospitalized | 1.60 ↓ | 0.64 ↓ | NA | all negative |
| P22 | GGO | Yes | Hospitalized | 5.64 | 1.41 | NA | all negative |
| P23 | GGO | Yes | Hospitalized | 4.61 | 1.14 | NA | all negative |
| P24 | GGO | Yes | Hospitalized | 5.20 | 1.31 | NA | all negative |
| P25 | GGO | Yes | Hospitalized | 7.56 | 0.73 ↓ | NA | all negative |
| P26 | GGO | Yes | Hospitalized | NA | NA | NA | all negative |
| P27 | GGO | Yes | Hospitalized | 4.40 | 1.11 | NA | all negative |
| P28 | GGO | Yes | Hospitalized | 4.84 | 1.22 | NA | all negative |
| P29 | GGO | Yes | Hospitalized | 3.31 ↓ | 1.07 ↓ | NA | all negative |
| P30 | GGO | Yes | Hospitalized | 6.64 | 2.07 | NA | all negative |
| P31 | GGO | Yes | Hospitalized | 4.94 | 1.78 | NA | all negative |
| P32 | GGO | Yes | Hospitalized | 4.13 | 1.41 | NA | all negative |
| P33 | GGO | Yes | Hospitalized | 9.62 ↑ | 0.62 ↓ | NA | all negative |
| P34 | GGO | Yes | Hospitalized | 9.91 ↑ | 0.52 ↓ | NA | all negative |
| P35 | GGO | Yes | Hospitalized | 9.14 | 1.89 | NA | all negative |
| P36 | GGO | Yes | Hospitalized | 8.26 | 1.87 | NA | all negative |
| P37 | GGO | Yes | Hospitalized | 5.52 | 0.92 ↓ | NA | all negative |
| P38 | GGO | Yes | Hospitalized | 8.55 | 0.33 ↓ | NA | all negative |
| P39 | GGO | Yes | Hospitalized | 5.54 | 1.94 | NA | all negative |
| P40 | GGO | Yes | Hospitalized | 3.92 | 1.49 | NA | all negative |
| P41 | GGO | Yes | Hospitalized | 6.89 | 1.20 | NA | all negative |
| P42 | GGO | Yes | Hospitalized | 5.66 | 0.59 ↓ | NA | all negative |
| P43 | GGO | Yes | Hospitalized | 13.00 ↑ | 0.20 ↓ | NA | all negative |
| P44 | GGO | Yes | Hospitalized | 3.51 | 1.66 | NA | all negative |
| P45 | GGO | Yes | Hospitalized | 5.66 | 1.86 | NA | all negative |
| P46 | GGO | Yes | Hospitalized | 10.89 ↑ | 1.21 | NA | all negative |
| P47 | GGO | Yes | Hospitalized | 2.90 ↓ | 1.29 | NA | all negative |
| P48 | Pleural bleb | Yes | Quarantine | 11.7 ↑ | 1.59 | NA | all negative |
| P49 | Lower-lobe pneumonia | Yes | Quarantine | 6.53 | 1.02 ↓ | NA | all negative |
| P50 | Pneumonia | Yes | Quarantine | 10.82 ↑ | 0.7 ↓ | NA | all negative |
| P51 | Secondary pulmonary tuberculosis | Yes | Quarantine | 13.93 ↑ | 1.0 ↓ | NA | all negative |
| P52 | Normal | Yes | Quarantine | 5.13 | 1.57 | NA | all negative |
| P53 | Fibrous stripes | Yes | Quarantine | 8.66 | 2.02 | NA | all negative |
| P54 | Subpleural nodules | Yes | Quarantine | 7.91 | 0.58 ↓ | NA | all negative |
| P55 | Normal | Yes | Health | 4.91 | 1.86 | 21.28 / 40.49 ↑ | all negative |
| P56 | Normal | Yes | Health | 7.68 | 1.63 | 1.29 / 2.62 | all negative |
| P57 | Emphysema | Yes | Health | 13.96 ↑ | 0.93 ↓ | NA | all negative |
| P58 | Fibrous stripes | Yes | Health | 8.09 | 1.04 ↓ | NA | all negative |
| P59 | Normal | Yes | Health | 5.69 | 1.12 | NA | all negative |
| P60 | Normal | Yes | Health | 9.27 | 3.66 ↑ | NA | all negative |
| P61 | Pneumonia | Yes | Health | 9.36 | 2.01 | 0.86 / 1.88 | Mycoplasma positive |
| P62 | Fibrous stripes | Yes | Health | 7.85 | 2.07 | 5.14 / 6.29 | all negative |
| P63 | Nodules | Yes | Health | 4.87 | 1.11 | NA | all negative |
| P64 | Lesions absorbed | Yes | Supposed convalescent^c^ | NA | NA | 28.31 / 128.14 ↑ | all negative |
| P65 | Lesions absorbed | Yes | Supposed convalescent | NA | NA | 23.23 / 115.22 ↑ | all negative |
| P66 | Lesions absorbed | Yes | Supposed convalescent | NA | NA | NA | all negative |
| P67 | Lesions absorbed | Yes | Supposed convalescent | NA | NA | 2.15 / 2.58 | all negative |
| P68 | Lesions absorbed | Yes | Supposed convalescent | NA | NA | 8.49 / 129.05 ↑ | all negative |
| P69 | Lesions absorbed | Yes | Supposed convalescent | NA | NA | 2.55 / 6.20 | all negative |
| P70 | Lesions absorbed | Yes | Supposed convalescent | 7.18 | 1.10 | 0.27 / 23.54 ↑ | Mycoplasma positive |
| P71 | Lesions absorbed | Yes | Supposed convalescent | 5.20 | 1.84 | 0.55 / 0.66 | all negative |
| P72 | Lesions absorbed | Yes | Supposed convalescent | NA | NA | NA | all negative |
| P73 | Normal | Yes | Supposed convalescent | 6.16 | 3.82 ↑ | NA | all negative |
| P74 | Lesions absorbed | Yes | Supposed convalescent | 4.79 | 2.18 | NA | all negative |
| P75 | Lesions absorbed | Yes | Supposed convalescent | NA | NA | 14.00 / 127.17 ↑ | all negative |
| P76 | Lesions absorbed | Yes | Supposed convalescent | NA | NA | 86.91 / 122.15 ↑ | all negative |
| P77 | Lesions absorbed | Yes | Supposed convalescent | NA | NA | 0.81 / 10.76 ↑ | all negative |

NA, not applicable

^a^ The chest CT image of COVID-19 is multiple patchy ground glass opacities (GGO) distribute along the subpleural area.

^b^ Including influenza A virus, influenza B virus, parainfluenza virus, rhinovirus, metapneumovirus and mycoplasma.

^c^ The supposed convalescents should be that: (1) temperature returned to normal for more than 3 days, and respiratory symptoms significantly improved; (2) chest CT imaging showed significant absorption of inflammation; (3) the nucleic acid test of respiratory pathogen was negative for two consecutive times, and the sampling interval should be at least 1 day, based on the official medical program.
